# Use of viscoelastic testing in the transfusion management of burn patients: a scoping review

**DOI:** 10.1007/s11239-025-03173-4

**Published:** 2025-09-11

**Authors:** Mariana Garay Álvarez, Giovanni Rodríguez Rojas, María Alejandra Triana Sutachan, Erwin Hernando Hernández Rincón

**Affiliations:** 1 Primary Care Physician, Universidad de La Sabana, Chía, Colombia; 2 Department of Family Medicine and Public Health , Universidad de La Sabana, Puente del Común University Campus , Km 7 Autopista Norte, Chía, Colombia

**Keywords:** Burns, Blood transfusion, Blood component transfusion, Thromboelastography, Viscoelastic testing, Scoping review (source: mesh NLM)

## Abstract

Severe burns cause complex hemostatic alterations that complicate transfusion management. Conventional coagulation tests (CCTs) have limitations in timely and accurately assessing these disorders. Viscoelastic tests (VETs), such as thromboelastography (TEG) and rotational thromboelastometry (ROTEM), allow for a dynamic and real-time evaluation of coagulation. A scoping review was conducted following the Arksey and O’Malley methodology and PRISMA-ScR guidelines. Studies from 2010 to 2025 in English or Spanish that evaluated the use of VETs in burn patients were included. Databases such as PubMed, Scopus, and ScienceDirect were analyzed. Eighteen studies were included, mostly narrative reviews and observational studies. ROTEM was the most reported test. VETs were mainly used at admission and intraoperatively. Evidence indicates that TEG and ROTEM outperform CCTs in sensitivity, specificity, and speed, allowing for reduced transfusions and detection of both hypocoagulability and hypercoagulability. VETs are promising tools for transfusion management in burn patients. Although their use is not yet standardized, they offer significant advantages over CCTs and promote more accurate clinical decisions. More studies are needed to support their systematic implementation.

**Graphical abstract:**

**Figure:**
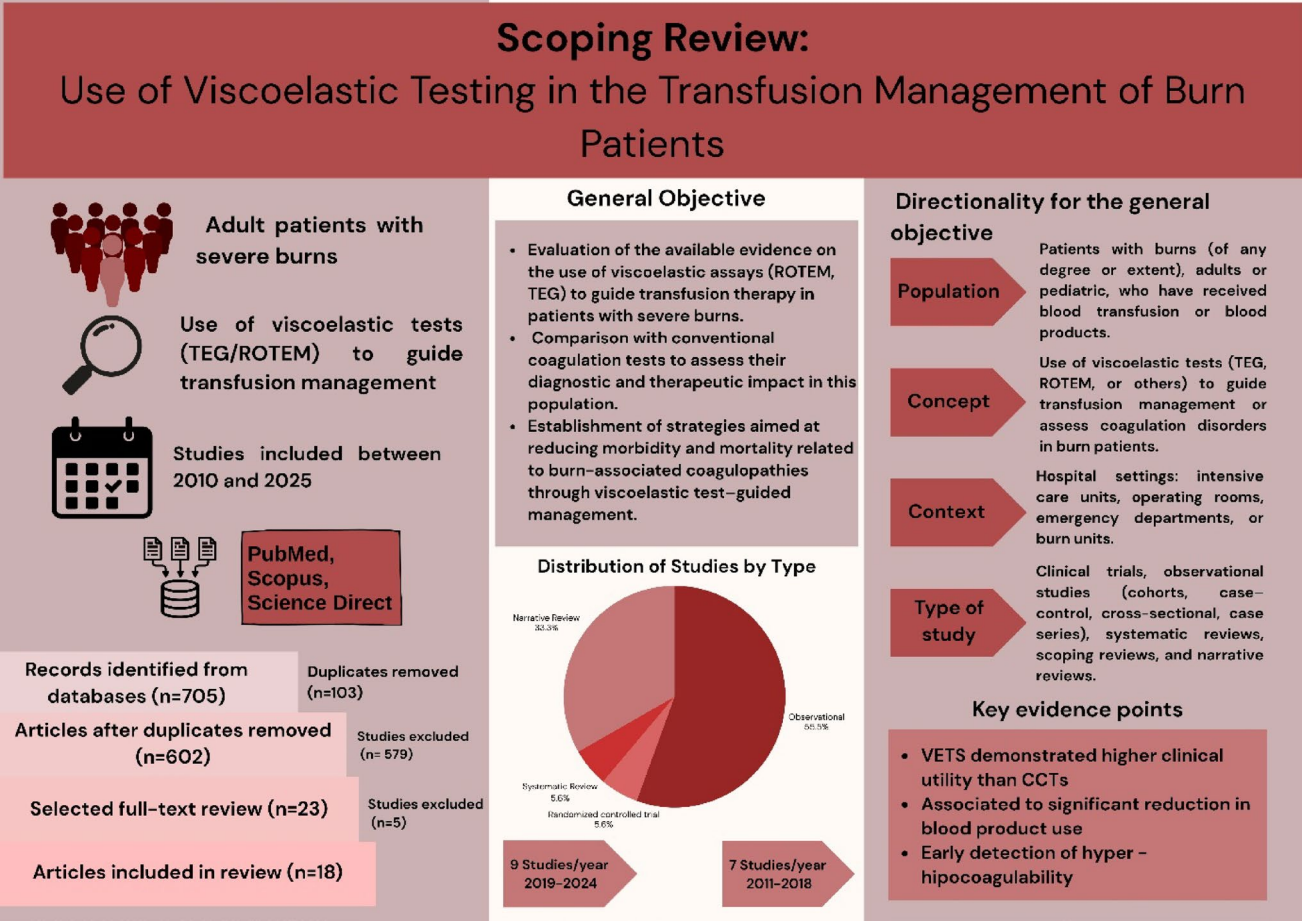
Scoping Review: Use of Viscoelastic Testing in the Transfusion Management of Burn Patients. TEG = thromboelastography; ROTEM = rotational thromboelastometry; CCTs = conventional coagulation tests

## Introduction

Severe burns represent a significant clinical burden and a complex challenge for medicine due to their high morbidity and mortality [1–4]. These patients experience profound pathophysiological alterations, including hemostatic dysfunction and hypovolemic shock, which are often exacerbated by surgical procedures such as burn excisions [5–7]. Moreover, massive blood loss is a major challenge during early excisional surgery and a predictor of mortality [2, 4]. Consequently, blood transfusions are a ubiquitous practice in the management of patients with extensive burns, being a critical need to maintain hemodynamic stability and counteract the frequently occurring anemia. However, the administration of allogeneic blood products carries inherent risks, including transfusion related to acute lung injury (TRALI), transfusion associated circulatory overload (TACO), immunosuppression, and increased infection rates, as well as prolonged hospital stays [4, 6, 8].

Traditionally, the assessment of coagulation in burn patients has relied on conventional coagulation tests (CCTs), such as prothrombin time (PT), activated partial thromboplastin time (aPTT), and plasma fibrinogen levels [3, 9]. While these tests are widely used, they present significant limitations in the dynamic context of burn injury. CCTs evaluate only a limited and static portion of the complex coagulation cascade and have not been validated to reliably predict bleeding in burn patients [3]. Moreover, their results may take up to an hour to become available, which restricts rapid decision making in acute situations where time is critical [9]. Often, CCTs may suggest a hypocoagulable state that does not always correlate with the clinical reality of bleeding; more importantly, they are ineffective in detecting hypercoagulability, a common and high risk condition for thromboembolic events in burn patients [3, 5, 10]. This inability to provide a global and real time picture of hemostasis underscores the need for more advanced diagnostic tools.

To better understand this limitation, it is important to consider the pathophysiology underlying coagulation disturbances in burn patients. Hypercoagulability in this population is caused by a coagulopathy induced by thermal injury, characterized by a procoagulant state that emerges within the first 48 h and may persist for days or weeks [1, 6]. This condition arises from an intense systemic inflammatory response that activates coagulation (via tissue factor expression), inhibits fibrinolysis (through increased levels of PAI-1), and reduces natural anticoagulants (such as thrombomodulin, protein C, and protein S) [4, 6, 7]. There is also an increase in fibrinogen, thrombin, and coagulation factors VIIa and VIII, along with platelet abnormalities and decreased antithrombin levels. Although fibrinolytic activity may be present, a hypofibrinolytic state predominates. Additional factors such as fluid resuscitation, hypothermia, and repeated surgeries further worsen hemostatic dysfunction, perpetuating the prothrombotic state [4, 6].

In response to these limitations, viscoelastic tests (VETs), such as thromboelastography (TEG) and rotational thromboelastometry (ROTEM), have emerged as dynamic and real time tools to evaluate the hemostatic function of whole blood [10, 11]. These tests offer a comprehensive view of the coagulation process, from initial clot formation to its stability and lysis, including the contribution of platelets and fibrinogen. Their main advantage lies in their ability to provide rapid results, enabling agile clinical decision making in emergency settings and the operating room [2, 9, 10]. Theoretically, VETs are more sensitive than CCTs in detecting burn related coagulopathies and have the potential to guide more targeted and factor specific administration of blood products, thereby reducing transfusion needs and associated adverse effects [3–5]. This has generated growing interest in their application in the acute management of severely burned patients.

Despite the theoretical advantages and growing interest, there is a lack of international consensus on the utility, standardized clinical application, and impact on outcomes of VETs in burn patients [2, 6, 7]. Existing studies often have small sample sizes and heterogeneous outcome measures, making it difficult to draw definitive conclusions and establish clear guidelines [5, 9, 10]. Furthermore, a considerable proportion of burn specialists still do not use VETs in their routine practice, and many are not even familiar with these techniques [12].

This variability in practice and the fragmented evidence highlights the urgent need for a systematic mapping of the existing literature to clarify their role. Therefore, the objective of this review is to synthesize the available literature on the use of VETs in the transfusion management of burn patients.

## Methods

A scoping review was conducted in June 2025 with the objective of answering the following research question: What is the available evidence on the use, effectiveness, and safety of VETs in the transfusion management of burn patients?, by mapping the relevant scientific literature. The protocol was registered in The Open Science Framework (OSF) [13], under the registration number: *osf.io/ep3d2*.

**Fig. 1:**
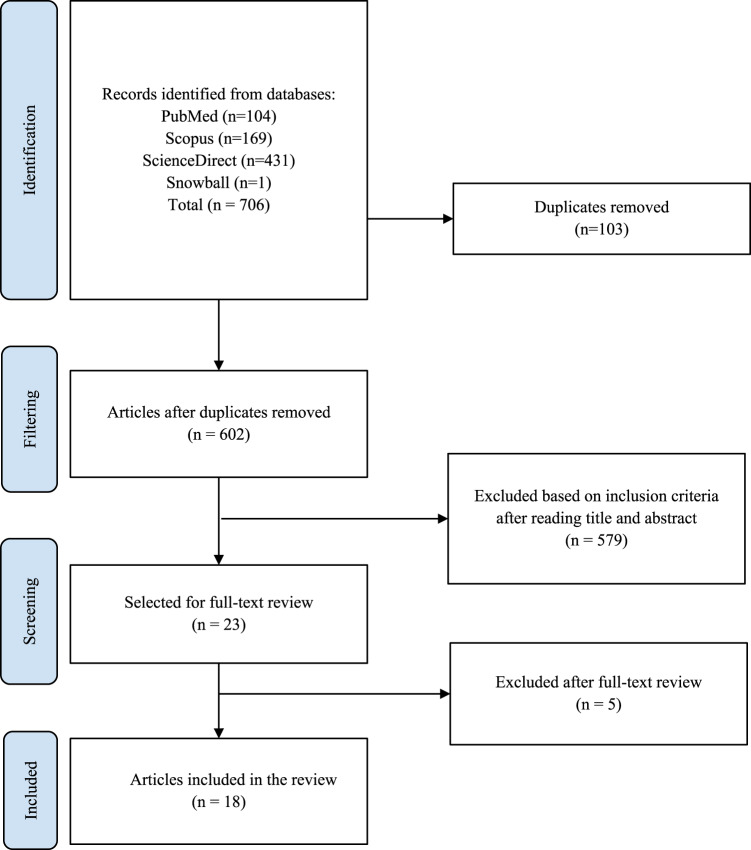
PRISMA flow diagram for the study selection process. Created using Shiny app [16, 17]

To ensure reproducibility and methodological rigor, the review followed the framework proposed by Arksey and O’Malley [14], complemented by the Joanna Briggs Institute (JBI) guidelines for scoping reviews [15]. Additionally, the Preferred Reporting Items for Systematic Reviews and Meta-Analyses extension for Scoping Reviews (PRISMA-ScR) was used as a guide for presenting the search process, study selection, and synthesis of findings (Fig. 1) [16, 17]. The following steps describe the development of this review.

### Identification of the research question and relevant studies

Medical Subject Headings (MeSH) and Health Sciences Descriptors (DeCS) such as “Viscoelastic Testing,” “Thromboelastography,” “Burns,” and “Blood Transfusion” were used, in combination with synonyms not recognized by the Virtual Health Library (VHL) and Boolean operators. The literature search was conducted in PubMed, ScienceDirect, and Scopus databases, selected for their broad coverage of biomedical and scientific literature, which allowed for a thorough and reliable exploration of the available evidence. Table 1 presents the search strategies and filters applied in each database, ensuring the reproducibility of the process.

**Table 1:** Search methods for each database

Database	Search strategy	Filters
PubMed	ALL FIELDS ((Burns) OR (Burn patients) OR (Major burns) OR (Severe burns) OR (Severe burn injury) OR (Burn injury)) AND ((Blood Transfusion) OR (Transfusion) OR (Blood component therapy) OR (Blood products) OR (Transfusion requirements) OR (Erythrocyte Transfusion)) AND ((Viscoelastic Testing) OR (Rotational Thromboelastometry) OR (TEG) OR (thromboelastography) OR (ROTEM) OR (thromboelastometry) OR (Viscoelastic assays) OR (viscoelastic test) OR (Blood Coagulation Tests) OR (Point-of-Care Testing) OR (blood clotting))	Publication date: 2010 − 2025 Full-text availability Language: Spanish and English
Scopus	ALL FIELDS ((Burns) OR (Burn patients) OR (Major burns) OR (Severe burns) OR (Severe burn injury) OR (Burn injury)) AND ((Blood Transfusion) OR (Transfusion) OR (Blood component therapy) OR (Blood products) OR (Transfusion requirements) OR (Erythrocyte Transfusion)) AND ((Viscoelastic Testing) OR (Rotational Thromboelastometry) OR (TEG) OR (thromboelastography) OR (ROTEM) OR (thromboelastometry) OR (Viscoelastic assays) OR (viscoelastic test) OR (Blood Coagulation Tests) OR (Point-of-Care Testing) OR (blood clotting))	Publication date: 2010 − 2025 Full-text availability Language: Spanish and English
Science Direct	(([Burns]) OR ([Burn] [patients]) OR ([Major] [burns]) OR ([Burn] [injury])) AND ([Blood] [Transfusion]) AND (([Viscoelastic] [Testing]) OR ([thromboelastography]) OR ([thromboelastometry]) OR ([Viscoelastic] [assays]))	Publication date: 2010 − 2025 Full-text availability Language: Spanish and English

Articles published between 2010 and 2025, written in English or Spanish, were included to identify the widest possible range of relevant evidence on the clinical utility of viscoelastic tests (VETs) in the transfusion management of burn patients. The decision to include studies from 2010 onward is justified by the remarkable rise in research, clinical validation, and global adoption of viscoelastic coagulation tests such as TEG and ROTEM, which became more accessible at the point of care and were widely recognized as superior tools for diagnosing and managing coagulopathy in this patient population [1, 7]. This period marked a turning point, evidenced by an exponential increase in clinical trials, improvements in clinical outcomes including reduced transfusion needs, enhanced survival, and cost-effectiveness, as well as the emergence of a new generation of automated devices that improved ease of use and reproducibility. Due to the limited number of publications initially identified through this strategy, a snowball search method was also implemented to expand the scope by reviewing the references of included articles to identify additional relevant studies.

## Selection criteria

The inclusion criteria for the studies were defined using the Population, Concept, and Context (PCC) strategy recommended by the Joanna Briggs Institute. In this review, the population corresponds to patients with burns of any degree of severity, the concept refers to the use of VETs (such as TEG or ROTEM) in transfusion management, and the context includes any clinical setting (burn unit, intensive care, operating rooms, among others) where such tests have been implemented.

Studies that evaluated the utility of these tests in transfusion decision-making, optimization of blood product use, identification of burn induced coagulopathies, or their impact on clinical outcomes were included. Various types of studies were considered valid, including clinical trials, observational studies, and systematic reviews. Editorials, letters to the editor, and commentaries were excluded.

Studies whose main focus was the use of VETs in non-burn related populations or in surgical contexts not pertinent to transfusion management in burn patients were also excluded. The full selection criteria applied in this review are detailed in Table 2.

**Table 2:** Selection criteria. Own elaboration

	Inclusion criteria	Exclusion criteria
Population	Patients with burns (of any degree or extent), adult or pediatric, who have received blood transfusion or blood products.	Studies on non-burn populations; studies exclusively in animals; studies on burn patients who did not require transfusion.
Concept	Use of viscoelastic tests (TEG, ROTEM, others) to guide transfusion management or assess coagulation abnormalities in burn patients.	Studies using viscoelastic tests unrelated to transfusion; studies evaluating only CCTs.
Context	Hospital settings: intensive care units, operating rooms, emergency departments, or burn units.	Prehospital care, non-clinical settings, or simulations; studies not related to thermal trauma or burns.
Type of Study	Clinical trials, observational studies (cohort, case-control, cross-sectional, case series), systematic reviews, scoping reviews, narrative reviews.	Editorials, letters to the editor, conference abstracts without full-text access, studies without original data or clear methodology, book chapters.

In addition to primary studies, this review intentionally included high-quality secondary reviews, such as systematic and narrative reviews, based on their methodological rigor and relevance to the research question. These reviews offered comprehensive syntheses of existing primary studies and provided critical contextualization of the clinical utility of VETs in burn patients, particularly in areas where individual studies were scarce, heterogeneous, or inaccessible. The decision not to extract and appraise each primary study included within these secondary sources was guided by several factors: (1) the risk of redundancy and data duplication in the synthesis; (2) the limitations in resource availability for re-assessing potentially large volumes of individual studies already critically appraised by experts; and (3) the added value of secondary reviews in highlighting overarching trends, expert consensus, and gaps in research. Furthermore, the use of these reviews aligns with the flexibility of the Arksey and O’Malley framework and JBI guidelines [14], which allow for the inclusion of diverse evidence types in scoping reviews, particularly when they contribute to a more holistic understanding of the topic and inform future research directions. Nonetheless, whenever possible, data from primary studies were prioritized and extracted directly if available and met the inclusion criteria independently.

## Study selection

After following the described methodology and importing the articles obtained from the search into RAYYAN, a tool designed to streamline the selection process in literature reviews [18], study selection was carried out in three sequential stages:

(1) An initial identification was performed considering only the titles and abstracts of the studies to identify potentially relevant articles, followed by the removal of duplicates.

(2) Subsequently, an eligibility assessment was carried out independently and blindly by two reviewers, who applied the previously established inclusion and exclusion criteria. In cases where discrepancies arose, these were resolved by a third reviewer, who made the final decision on the inclusion or exclusion of the disputed studies.

(3) Finally, the selected articles underwent a full text review for final evaluation, assessing their specificity, methodological rigor, and direct relevance to the use of VETs in the transfusion management of burn patients. This rigorous process ensured the quality and consistency of the selected studies, confirming their relevance to the proposed scoping review.

## Data collection, summary, and presentation of results

Once the full-text review was completed and articles were selected, systematic data extraction was performed using a synthesis table that allowed for structured organization and visualization of information. This table includes first author and year of publication, a content summary, type of study, types of VETs used, timing of the evaluation, and the country where the study was conducted (Table 3).

**Table 3:** Summary of the documents included in the review

First author, year	Types of viscoelastic tests		Evaluation moment	Country	Summary	TBSA (%)
*Descriptive Cross-sectional Studies*						
Roggan, 2023 [1]	ROTEM		Admission, intraoperative and follow-up	Multinational	Survey study on trends in coagulation and temperature management in burns; increased use of point-of-care testing and factor-based management; and universalization of normothermia protocols.	No
Lavrentieva, 2016 [12]	TEG, ROTEM		According to clinical practices (not standardized)	Multinational	Survey of burn ICU physicians on diagnostic and treatment practices for coagulopathy. 70.8% used standard tests, and only a minority used TEG/ROTEM. There is no consensus on definition or management.	No
*Systematic Reviews*						
Marsden, 2017 [2]	ROTEM, TEG		Variable according to the studies included	Multinational	Systematic review concludes that TEG and ROTEM are superior to standard tests (PT/APTT) for identifying coagulopathies in burn patients; more robust evidence is needed.	25–30%
*Observational studies*						
Jian, 2023 [3]	Retrospective observational study	TEG	Admission	China	Patients with early burns showed a hypercoagulable state on TEG: decreased R and K values, increased MA, CI, and alpha angle. Severity was associated with greater abnormalities. TEG had predictive value for complications such as bleeding and kidney or lung injury.	32.6 ± 10.4%
Wiegele, 2019 [5]	Prospective cohort study	ROTEM	Days 1 (post trauma), 2 (post excision), 7 and 14 post-admission	Austria	The hypercoagulable state was assessed in patients with severe burns using TGA, ROTEM, and conventional tests. TGA showed increased thrombin peak and velocity index; ROTEM showed increased clot firmness and alpha angle. Conventional tests did not detect abnormalities.	38.5 ± 19.8%
Sahli, 2021 [6]	Retrospective cohort study	ROTEM	Day of admission, before the first surgery, and upon discharge from the ICU	Swiss	Blood product transfusion was compared before and after implementing a goal-directed, factor-based coagulation algorithm. A significant reduction in transfusions and fibrinogen use was observed, with a trend toward lower mortality.	32.5 ± 17.7%
Huzar, 2017 [9]	Retrospective observational study	TEG	Admission	USA	Admission rTEG predicted resuscitation volumes, transfusions, and mortality. 60% were in a hypercoagulable state, and 24% were in a hypocoagulable state. ACT ≥ 128 s was associated with increased fluid requirements, and angle < 60° with increased mortality.	38.0 ± 17.6%
Mathew, 2024 [20]	Prospective observational clinical study	TEG, ROTEM	Admission, pre-plasma, post 1 unit, post-resuscitation (up to 48 h)	USA	We evaluated whether plasma resuscitation altered the coagulation profile. No prothrombotic changes were observed with the use of plasma according to TEG and ROTEM at different time points. There were no differences in mortality, burn size, or inhalation.	41.3 ± 19.5%
Pidcoke, 2015 [21]	Prospective observational clinical study	ROTEM	Intraoperative	USA	An observational study that characterized transfusion during burn excisions found that the transfused products failed to adequately restore hemostasis, even with high platelet ratios.	40.3 ± 18.8%
Schaden, 2012 [23]	Prospective observational clinical study	ROTEM	Admission, 12 h, 24 h and 48 h later	Austria	Prospective study showing increased fibrinogen function (FIBTEM MCF) and fibrinogen concentration 48 h after severe burns; significant correlation between the two parameters.	42.55 ± 21.81%
Li, 2022 [24]	Retrospective observational study	TEG	Diary from admission to death (within 10 days)	China	Retrospective analysis of R-TEG data in patients with severe burns (TBSA ≥ 60%). It showed progressive alterations in coagulation and fibrinolysis culminating in DIC. TEG allowed early detection of coagulation dysfunction.	90.0 ± 5.6%
*Narrative review*						
Palmieri, 2019 [4]	TEG, ROTEM		Intraoperative and ICU	USA	Review of transfusion strategies for burn patients. It concludes that a restrictive approach is safe and that TEG/ROTEM are useful for guiding hemostatic product transfusion.	≥ 20%
Welling, 2018 [7]	TEG, ROTEM		During escharectomy	Denmark	Systematic review of intraoperative hemorrhage monitoring and resuscitation strategies in burn surgery. It concludes that TEG and ROTEM reduce transfusions and allow for targeted therapies, but randomized controlled trials are lacking.	≥ 20%
Wiegele, 2017 [10]	ROTEM, TEG		Variable; emphasizes intraoperative use and hemorrhage	Austria	Review on the use of viscoelastic testing in burn patients; suggests that ROTEM and TEG help guide management and detect hypercoagulability; consensus and standardization are still lacking.	≥ 20%
Tantry, 2023 [11]	TEG, ROTEM		Perioperative/peri-intervention	Multinational	Review of the role of viscoelastic tests in assessing platelet function and coagulation in periprocedural settings. The potential of VHs to evaluate coagulation dynamics and guide therapeutic decisions is highlighted.	20–30%
Ball, 2020 [19]	TEG, ROTEM		Acute phase (< 48 h), post-resuscitation phase (> 48 h), intraoperative, convalescence	USA	Analyzes coagulopathy due to burns, its mechanisms and evolution, highlighting the value of viscoelastic tests (TEG, ROTEM) and their advantages over conventional methods, as well as their main complications.	30–33%
Brazier, 2011 [22]	ROTEM		Not specified, suggested for intraoperative use	United Kingdom	Literature review on blood loss in burn surgery; proposes that thromboelastometry could be useful in the future for developing transfusion protocols.	15–70%
*Randomized Controlled Trial*						
Schaden, 2021 [8]	ROTEM		Preoperative, intraoperative and postoperative until the next day	Austria	Prospective randomized study comparing a ROTEM-guided bleeding management algorithm versus standard management. The algorithm significantly reduced allogeneic transfusions.	33.78 ± 14.56%

Additionally, to systematize the interpretation of findings and establish a structured comparison between CCTs and VETs, a matrix of criteria was developed to classify the reported utility in each study. This categorization was organized into three levels of utility (high, medium, and low), defined based on the clinical applicability of the tests, their impact on transfusion decision-making, correlation with relevant clinical outcomes, and their incorporation into care protocols. Table 4 presents these criteria for each test type, specifically distinguishing between CCTs and VETs to ensure accurate and reproducible evaluation of the included evidence. These criteria were then applied to all selected studies, classifying each of them. This information was systematized which comparatively displays the relative utility of both test types in the context of transfusion management for burn patients.

**Table 4:** Comparison between conventional coagulation tests and viscoelastic tests in burn patients

Level of Usefulness	Description	Conventional Coagulation Tests (CCT)	Viscoelastic Tests (VET)
High	- Directly guide transfusion decisions. - Associated with better clinical outcomes. - Used as the main tool in transfusion algorithms. - Reduction in transfusions or complications. - Rapid results with immediate clinical application	None	Roggan [1], Palmieri [4], Wiegele [5], Sahli [6], Welling [7], Schaden [8], Huzar [9], Tantry [11], Ball [19], Li [24]
Medium	- Identify basic abnormalities but do not guide specific decisions. - Limited usefulness in dynamic scenarios. - Indirect correlations with outcomes - Detect coagulopathies or hypercoagulability but do not change management. - Useful findings but not validated in clinical protocols.	Roggan [1], Jian [3], Palmieri [4], Sahli [6], Schaden [8], Huzar [9], Lavrentieva [12], Mathew [20]	Marsden [2], Jian [3], Wiegele [10], Lavrentieva [12], Mathew [20], Pidcoke [21], Schaden [23]
Low	- Do not correlate with the patient’s actual clinical condition. - Normal results despite clinical abnormalities. - Unreliable in burn patients. - Descriptive use without clinical implications. - Contradictory or uninterpretable results. - Insufficient evidence to define usefulness.	Marsden [2], Wiegele [5], Welling [7], Wiegele [10], Tantry [11], Ball [19], Pidcoke [21], Brazier [22], Schaden [23], Li [24]	Brazier [22]

## Results

### General description of included studies

The initial search identified 706 articles. After removing 103 duplicates, 602 titles and abstracts were screened, of which 579 were excluded for not meeting the inclusion criteria. Full text review was conducted on 23 articles, and 5 were excluded. Ultimately, 18 studies were included in this review. The most frequent study types were narrative reviews (*n* = 6, 33.3%), followed by prospective observational clinical studies (*n* = 3, 16.7%) and retrospective observational studies (*n* = 3, 16.7%). A smaller proportion consisted of cross-sectional descriptive studies (*n* = 2, 11.1%), as well as systematic reviews (*n* = 1, 5.6%), prospective cohort studies (*n* = 1, 5.6%), retrospective cohort studies (*n* = 1, 5.6%), and randomized controlled trials (*n* = 1, 5.6%).

Regarding the viscoelastic monitoring tools used or primarily analyzed, ROTEM was the most frequently reported, either alone or in combination, appearing in 12 studies (66.7%). TEG was mentioned in 9 studies (50%), while both methods, ROTEM and TEG, were used together in 10 studies (55.6%). When broken down by usage type, ROTEM was exclusively reported in 6 studies (33.3%), TEG in 2 studies (11.1%), and both in combination in 10 studies (55.6%).

The evaluation timing of viscoelastic monitoring varied among the included studies. Most measurements were performed at admission or initial presentation (*n* = 5, 27.8%), highlighting the utility of ROTEM/TEG as rapid assessment tools in critically burned patients, specifically in the early phases of burn injury, where coagulopathy may not yet be clinically evident and there is a complex inflammatory and prothrombotic response. Intraoperative use was also common (*n* = 4, 22.2%), especially in contexts of eschar excision or hemorrhage control frequently in surgical burn management when they are often subjected to high-impact procedures such as grafts and flaps. Some studies implemented postoperative or serial follow up protocols (*n* = 3, 16.7%) and monitoring during the acute resuscitation phase (*n* = 3, 16.7%), allowing dynamic hemostatic trend observation. Two studies performed sequential evaluations at defined intervals (*n* = 2, 11.1%), and one used variable or non-standardized time points (*n* = 1, 5.6%).

In terms of geographic origin, most included studies were conducted in high-income countries, with a predominance of research from the United States (*n* = 5, 27.8%) and Austria (*n* = 4, 22.2%). Additionally, a significant number of studies were multinational (*n* = 4, 22.2%), suggesting a growing global interest in standardizing the use of viscoelastic monitoring in burn patients. China contributed two studies (11.1%), and each of the following countries contributed one study (5.6%): the United Kingdom, Switzerland, and Denmark.

On the other hand, based on the reviewed evidence in Table 3 total burn surface area (TBSA) emerges not only as a burn severity marker but also, as a useful criterion for deciding whether viscoelastic testing (TEG/ROTEM) should be implemented at admission. Most observational studies demonstrate that patients with TBSA ≥ 30% already present measurable coagulation abnormalities on TEG/ROTEM upon arrival, despite normal standard laboratory tests, supporting early detection of hyper or hypocoagulable states. Narrative and systematic reviews commonly suggest TBSA thresholds between 20 and 30% as triggers for incorporating viscoelastic testing in the early assessment of burn patients, with TBSA ≥ 60% representing a critical subgroup where these assays become essential given the rapid evolution toward coagulopathy or DIC.

## Utility of vets in transfusion management

The reported utility of CCTs and VETs varied among the included studies (Table 4). Overall, most articles classified the utility of VETs as high (*n* = 10, 55.6%), suggesting strong clinical value, especially in complex transfusion management scenarios unique to burn patients, who often result in unpredictable coagulation changes and extensive tissue damage, which can significantly alter coagulation pathways. A significant number of studies were classified with moderate utility (*n* = 7, 38.9%), reflecting partial applicability or context dependent usefulness. Only one study reported low utility (*n* = 1, 5.6%), indicating significant limitations in guiding transfusion decisions.

In burn patients, VETs have been consistently recognized as key tools for dynamic coagulation monitoring in burn patients [10, 11]. By analyzing whole blood, these tests offer a comprehensive assessment of hemostasis, from clot formation to stabilization and lysis. This capacity is valuable in burn care, where coagulation disturbances are variable due to systemic inflammation, capillary leak and tissue necrosis. VETs allow simultaneous evaluation of both coagulation factor contribution and platelet function, providing valuable information for clinical decision-making [2].

Furthermore, the rapid availability of results (within 10 to 20 min) determines their utility as point-of-care (POC) tools, particularly in early hours post-burn, when rapid decision making prevents coagulopathic bleeding or unrecognized thrombotic risk, this becomes critical. Without timely identification and targeted intervention patients are at increased risk of hemorrhage during debridement or thromboembolic events. In surgical procedures involving burn patients, often associated with significant bleeding, these tests have proven effective in guiding rational administration of blood components and reducing transfusion requirements [1, 2, 4, 5, 7, 10, 11, 19].

A randomized prospective study of 30 severely burned patients undergoing excision and grafting showed that a ROTEM based resuscitation algorithm reduced cumulative blood product use by more than half compared to standard care [8]. Moreover, the implementation of factor guided, POC based coagulation management algorithms within patient blood management programs has contributed to reducing allogeneic blood transfusions and associated adverse effects [1, 6]. These findings are especially relevant in burn patients, who are frequently subjected to multiple surgeries and prolonged ICU stays. For instance, this trend has been particularly notable in European burn centers, where a progressive increase between 2016 and 2021 was observed in the use of fibrinogen concentrate, prothrombin complex concentrate, factor XIII, and recombinant factor VIIa [1].

VETs have also demonstrated utility in detecting hypercoagulable states, which are clinically relevant due to the high incidence of thromboembolic events in burn patients [3, 5, 10, 19]. In this regard, a study using rapid thromboelastography (rTEG) at hospital admission showed that a hypocoagulable state was associated with a fivefold increase in the likelihood of requiring supranormal resuscitation, as well as increased mortality [9].

Finally, one observational study indicated that plasma resuscitation in burn patients was safe and not associated with thrombotic complications or prothrombotic or fibrinolytic alterations [20]. This supports the value of VETs in accurately monitoring the hemostatic response to transfusion and guiding safer, targeted interventions in early burn care.

## Comparison with CCTs

Regarding the utility of CCTs, the studies included revealed a trend toward limited perception (Table 4). Most articles were classified as having low utility (*n* = 11, 61.1%), suggesting a restricted capacity to appropriately guide transfusion management in severely burned patients. A smaller proportion was categorized with moderate utility (*n* = 7, 38.9%), reflecting partial or context dependent clinical value. No study reported high utility, highlighting the limitations of these tests in highly complex clinical scenarios.

The limited utility of CCTs such as PT, aPTT, and platelet count for adequately assessing hemostasis in burn patients has been consistently emphasized [2–4, 7–10, 12]. These tests often fail to reflect the functional changes occurring in coagulation dynamics after thermal injury [5, 19].

In several studies, CCTs have shown normal-range results even in the presence of a hypercoagulable state identified via VETs [5, 19]. This discordance can lead to misinterpretations, such as suspecting a bleeding tendency when the patient is actually in a procoagulant state [10]. This discrepancy is explained by the fact that CCTs analyze only plasma and are terminated at the formation of initial fibrin strands, whereas VETs assess whole blood and allow for evaluation of clot formation, quality and stability, providing a more comprehensive and representative view of the in vivo coagulation process [3, 8, 10].

Nonetheless, studies have demonstrated that techniques such as TEG and ROTEM are more sensitive than CCTs for detecting burn induced coagulopathy (BIC) [2, 3]. In particular, TEG has been reported to more accurately detect hypercoagulability than plasma-based tests. Despite these advantages, a survey revealed that 70.8% of burn management specialists continue to use CCTs as the primary diagnostic tools to detect BIC, and a considerable proportion are unfamiliar with viscoelastic techniques [12, 19].

Additionally, it was observed that resuscitation with blood products in patients with extensive burns or soft tissue injuries does not always guarantee effective hemostasis, as transfused components such as platelets may present functional impairments acquired during storage or due to the patient’s inflammatory environment. These dysfunctions, which affect clot quality, are not detected by CCTs [2, 3, 10, 11, 21, 22].

## Discussion

### Limitations of CCTs in burn patients

Traditionally, patients with extensive burns have been evaluated using CCTs, such as PT and PTT, to guide transfusion management. However, in burns affecting more than 30% of the total body surface area, a systemic inflammatory response is triggered, activating the coagulation cascade and potentially resulting in disseminated intravascular coagulation (DIC). Burn injuries induce a complex and dynamic coagulopathy that often escapes detection by conventional methods. Typical findings in burn trauma include prolonged PT and PTT, decreased platelet count, variable fibrinogen levels, and increased D-dimer. These results may vary depending on burn severity and may lead to microvascular thrombosis contributing to organ dysfunction. However, these tests are insufficient due to their low sensitivity, especially in detecting subtle hemostatic alterations. Moreover, it is common for these tests to return normal results despite significant coagulation disorders, or they may be falsely interpreted as indicative of bleeding risk, potentially leading to unnecessary interventions that worsen the patient’s condition [3, 10, 23].

This discrepancy is explained by the fact that CCTs analyze only plasma and are terminated at the formation of initial fibrin strands, whereas VETs assess whole blood and allow for evaluation of clot formation, quality and stability, providing a more comprehensive and representative view of the in vivo coagulation process [3, 8, 10].

### Role of vets as dynamic tools

Given this scenario, the use of VET such as TEG and ROTEM has gained relevance, as these allow a more integrated and dynamic evaluation of the coagulation process. These tests have proven particularly useful in the context of severe burns, where a biphasic pattern has been described: an initial hypercoagulable state followed by hypocoagulability in later stages. This evolution is influenced by multiple factors, including burn depth, presence of inhalation injury, hypothermia, hemodilution during the resuscitation phase, and multiple required surgical interventions [2, 10].

Burn-induced coagulopathy exhibits a biphasic evolution, with a predominant hypercoagulable state during the first 48–72 h, attributed to the massive release of proinflammatory cytokines (e.g., IL-6, TNF-α), endothelial activation, tissue factor expression, and increased synthesis of acute-phase proteins like fibrinogen. This is often followed by a progressive hypocoagulable phase due to platelet dysfunction, coagulation factor consumption, hyperfibrinolysis, and dilutional effects from fluid resuscitation. The intensity and duration of each phase may vary according to factors such as TBSA, depth of injury, inhalation injury, and surgical interventions.

Endothelial injury and glycocalyx disruption also play a central role in the prothrombotic environment, contributing to activation of the contact pathway and impairment of natural anticoagulant mechanisms (e.g., antithrombin, protein C). These changes often go undetected by routine CCTs, further supporting the need for dynamic assessment tools. Moreover, in patients with extensive burns, complications such as sepsis and DIC are observed during the clinical course, specifically in patients with prolonged intensive care stays. For instance, DIC may develop as a maladaptive response, with the widespread microvascular thrombosis followed by consumption of clotting factors and platelets, leading to a paradoxical bleeding tendency. These events are accompanied by rapid, unpredictable fluctuation in coagulation status, TEG and ROTEM track the dynamic changes seen in septic and criticality burn patients [10].

During extensive surgical excisions, blood loss can be significant, necessitating transfusion or coagulopathy correction. In this context, VETs have emerged as valuable point-of-care tools, both intraoperatively and in intensive care units, facilitating rapid and personalized hemostatic decisions. While their use is well established in scenarios such as cardiac surgery or liver transplantation, their application in burn patients is expanding as a guide for correcting coagulopathy via appropriate factor replacement or transfusion of blood products [1, 2].

By analyzing whole blood, these tests offer a comprehensive assessment of hemostasis, from clot formation to stabilization and lysis. This capacity allows simultaneous evaluation of both coagulation factor contribution and platelet function, providing valuable information for clinical decision-making [2].

Furthermore, the rapid availability of results (within 10 to 20 min) determines their utility as POC tools, particularly valuable in acute bleeding scenarios requiring urgent clinical decisions. In surgical procedures involving burn patients, often associated with significant bleeding, these tests have proven effective in guiding rational administration of blood components and reducing transfusion requirements [1, 2, 4, 5, 7, 10, 11, 19].

Intraoperative bleeding may be extensive and secondary to undetected or uncorrected coagulopathy. However, recent recommendations advocate for a restrictive use of blood products, particularly allogeneic transfusions, due to their associated complications. Traditionally, a 1:1:1 transfusion ratio of red blood cells, platelets, and plasma is recommended, along with direct replacement of coagulation factors [1]. Nonetheless, most institutions lack established protocols for using VETs as point-of-care coagulation tests, which are defined as bedside diagnostic tools with the advantage of shorter turnaround times and ease of execution [10]. In this regard, ROTEM has been identified as the most widely available and commonly used test, particularly during surgical procedures, with estimated usage above 60% according to studies. However, its use upon emergency department admission remains limited, despite a growing trend in VET adoption over the years [1, 4].

Despite transfusion management recommendations, studies have shown that blood component replacement often does not follow a balanced-component approach, with lower use of platelets and plasma compared to red blood cells. Even when resuscitation involves blood products, a study using ROTEM showed that transfused products often resulted in abnormally weak clots, with clot strength below the reference range, although slight improvement was observed with products containing platelets. Therefore, assumptions regarding the hemostatic function of transfused products should be avoided, as these products, particularly platelets, may exhibit functional deficiencies, and tests like ROTEM may be critical for assessing clot quality [21].

One major coagulation issue that may go unnoticed is hypercoagulability, which can appear as early as the first week after burn injury, associated with increased fibrinogen synthesis. During the first 24 h post-burn, elevated levels of tissue type plasminogen activator inhibitor-1 have been observed [10]. In this regard, the ROTEM parameter FIBTEM MCF (maximum clot firmness) has shown correlation with fibrinogen levels measured via the Clauss method, indicating that VETs can more sensitively detect early procoagulant states than conventional methods. The time advantage is also significant, with approximately 20 min for ROTEM compared to one hour for CCTs, potentially reducing adverse events and costs by minimizing the unnecessary use of platelet and FFP concentrates through individualized coagulation management [23].

As previously mentioned, the hypercoagulable state may persist for up to two weeks post injury. Studies evaluating thrombin generation in thermal injury patients [22], using TGA parameters such as peak thrombin and velocity index, found significantly elevated levels peaking one week after the burn and returning to near normal levels by the second week. ROTEM also showed signs of hypercoagulability, such as increased alpha angle and MCF. However, CCT findings like PT, aPTT, and platelet count, remained within reference ranges and failed to detect the hypercoagulable state. This occurs because CCTs only assess the initial phase of thrombin generation, missing approximately 95% of thrombin produced later. Nonetheless, TGA is currently not widely available and is associated with high costs; thus, tests like ROTEM may offer substantial clinical utility [5].

Regarding the use and availability of rTEG, studies in patients with > 60% TBSA burns have evaluated changes in parameters such as alpha angle, maximum amplitude (MA), activated clotting time, and kinetic time, which significantly decreased, indicating increasing hypocoagulability and platelet dysfunction. The MA in rTEG is particularly important for reflecting platelet function. rTEG provides a basis for timely therapeutic interventions, such as targeted blood product transfusion, to counteract tissue hypoperfusion and restore coagulation balance [24].

### Diagnostic variability and challenges in detecting DIC in burn patients

DIC is a consumptive coagulopathy characterized by systemic activation of coagulation, leading to platelet and clotting factor depletion, severe hemorrhage, and thrombotic obstruction of the microcirculation, which may promote organ dysfunction in burn patients. Although clinically overt DIC appears to occur in only a minority, its association with mortality is significant: 88.9% of deceased patients presented with DIC at the time of death, suggesting a decisive role in outcomes. Sepsis, present in 55.6% of cases, further contributes by inducing secondary DIC and septic shock, worsening hemostatic dysfunction [24].

Prevalence rates vary markedly by diagnostic criteria. Approximately 30% of severe burns are estimated to develop DIC [23], whereas retrospective series using the International Society of Thrombosis and Haemostasis (ISTH) criteria have reported incidences up to 91% in patients with > 25% TBSA burned. Conversely, other studies report much lower incidences (0.09% in the general population and 0.66% in TBSA > 20%), reflecting substantial heterogeneity in definition [10].

D-dimer and fibrin degradation product (FDP) levels are significantly higher in severe versus mild or moderate burns, suggesting increased thrombotic risk and early DIC [3]. Viscoelastic hemostatic assays (VHA) such as R-TEG^®^ show that coagulopathy is progressive: hypocoagulability parameters (ACT, K, α, MA) are altered early post-injury, and by the second week, hyperfibrinolysis (elevated LY30) is frequently observed, consistent with progression toward DIC. These abnormalities can precede clinical diagnosis or fulfillment of ISTH criteria, underscoring the value of VHA for dynamic monitoring [23].

R-TEG^®^ findings can also guide targeted transfusion strategies (e.g., fresh frozen plasma for prolonged ACT, platelets for reduced MA, antifibrinolytics for elevated LY30). However, in extensive burns with advanced coagulopathy, VHA parameters may not normalize after surgery or transfusion, highlighting the severity of the disorder and the need for VHA-based transfusion protocols [23]. In this setting, tranexamic acid administration should be guided by VHA results when available, following trauma standards [10].

In early thermal injury, additional mechanisms, including hemodilution from large-volume resuscitation, hypothermia due to skin barrier loss, and shock-associated hypoperfusion, also contribute to coagulopathy, typically manifested as prolonged PT and aPTT [10]. The heterogeneity in DIC presentation and diagnosis in burn patients highlights the need for standardized, dynamic approaches; VHAs represent a promising tool for early detection and optimized blood product use in this high-risk population [19].

### Balancing thrombosis and bleeding: anticoagulation in the burn population

It is well known that both procoagulant and anticoagulant states can occur in various conditions, including burn patients, who are at risk of developing DIC. A strong correlation has been demonstrated between the use of VHA and both organ dysfunction and ICU survival. In critically ill patients, VHA can also be used to guide anticoagulation monitoring when aPTT and anti-Xa levels are inconclusive, offering an alternative for dose adjustment [10].

Anticoagulant management in burn patients has become increasingly complex due to the hypercoagulability and hypofibrinolysis that characterize burn-induced coagulopathy. This prothrombotic profile, combined with endothelial injury and venous stasis, increases the risk of deep vein thrombosis (DVT) and venous thromboembolism (VTE). However, the true incidence of these thrombotic events varies widely across studies (0.2–25%), partly because of methodological differences such as the inclusion of autopsy studies or systematic ultrasound screening. The reported incidence of pulmonary embolism is also highly variable, ranging from 0.001 to 3.3%.

The use of prophylactic anticoagulation remains controversial. While some studies have not demonstrated a reduction in VTE incidence and raise concerns about bleeding risk, others show clear benefits in reducing DVT without significant hemorrhagic complications. Moreover, pharmacodynamic studies have shown that standard enoxaparin dosing may be insufficient in this population, further complicating clinical decision-making [19]. These conflicting data highlight the need for more precise guidelines tailored to the pathophysiological context of burn patients. In this regard, viscoelastic testing could provide an additional tool to guide the safe and effective use of anticoagulants, personalizing treatment according to the patient’s real-time coagulation status [5, 19].

In patients with severe burns, red blood cell transfusion is initiated early to maintain hemoglobin levels between 5.6 and 6.2 mmol/L due to the risk of intraoperative bleeding. Fresh frozen plasma is administered in cases of active bleeding and laboratory evidence of INR > 1.5 and prolonged aPTT. Patients receiving therapeutic anticoagulation required 5–8 times more red blood cells and 2–4 times more plasma than those on prophylactic anticoagulation. Although no statistically significant association between transfusion volume and mortality was found, there was a trend toward improved survival with higher red blood cell transfusions and, conversely, a trend toward increased mortality with larger plasma volumes [7].

### Advantages and challenges of ROTEM/TEG use

Finally, the use of VETs as rapid diagnostic tools for identifying coagulopathy and guiding the use of blood products during resuscitation in burn patients provides significant advantages over CCTs: Response times of 10–20 min, higher sensitivity and specificity, and the ability to reduce unnecessary blood component use, thereby lowering the risk of adverse events from massive transfusions. Nevertheless, implementation poses challenges such as technological availability, the need for periodic calibration, and clinical staff training for proper interpretation and application [2, 3].

### Limitations

Several limitations must be considered when interpreting the findings. As this is a scoping review, it does not aim to establish causal relationships or issue clinical recommendations. The available evidence is scarce and of low quality, consisting mainly of small, heterogeneous studies, most of which are observational and at high risk of bias. In the case of surveys, results are based on subjective perceptions, with low representativeness and without a multidisciplinary approach, which further limits their applicability.

## Conclusion

VETs represent dynamic and sensitive diagnostic tools that address the limitations of CCTs in the transfusion management of burn patients. Their implementation enables a comprehensive assessment of hemostasis, supports timely clinical decision-making, and may reduce the unnecessary use of blood products. However, challenges remain regarding standardization, availability, and clinical staff training, underscoring the need for more robust studies and tailored clinical protocols for this population.

## Data Availability

No datasets were generated or analysed during the current study.
